# Editorial: Obesity, Smoking, and Fatty Liver Disease

**DOI:** 10.3389/fendo.2018.00001

**Published:** 2018-01-18

**Authors:** Amiya P. Sinha-Hikim, Sushil K. Mahata

**Affiliations:** ^1^Department of Internal Medicine, Charles R. Drew University of Medicine and Science, Los Angeles, CA, United States; ^2^David Geffen School of Medicine, University of California, Los Angeles, Los Angeles, CA, United States; ^3^VA San Diego Healthcare System (VHA), San Diego, CA, United States; ^4^Department of Medicine, University of California, San Diego, La Jolla, CA, United States

**Keywords:** obesity, diabetes, immunometabolism, cardiomyopathy, fatty liver disease, insulin sensitivity, RNA splicing, skeletal muscle regeneration

**Editorial on the Research Topic**

**Obesity, Smoking, and Fatty Liver Disease**

Obesity is on the rise worldwide and is doing so at an alarming rate. Obesity constitutes a major risk factor for diabetes and associated disorders like altered innate (neutrophils, dendritic cells, macrophages, mast cells, and eosinophils) and adaptive (B and T lymphocytes) immune cell responses to metabolism, diabetic cardiomyopathy (DCM), cardiovascular dysfunctions, non-alcoholic fatty liver disease (NAFLD), and certain forms of cancer. The health risks associated with obesity are further exaggerated by smoking. This research topic consisting of 10 articles (9 reviews and 1 original) provides a comprehensive assessment of the impact of obesity on immunometabolism, cardiac functions, the connections of nicotine to NAFLD, the expression of hepatic carcinoembryonic antigen-related cell adhesion molecule 1 (CEACAM1), the role chromogranin A (CgA) and its peptides pancreastatin (PST) and catestatin (CST) in insulin sensitivity, the skeletal muscle regeneration, and the alternate RNA splicing.

The first article by Mayoral Monibas et al. discusses the identification and contribution of hepatic non-parenchymal cells such as resident Kupffer cells (KCs), recruited monocyte-derived hepatic macrophages (RHMs), resident innate lymphocytes or natural killer cells, and fat storing hepatic stellate cells (HSCs) in the development of NAFLD, non-alcoholic steatohepatitis (NASH), and fibrosis through the use of cell surface markers. The authors underscore the polarization of hepatic macrophages from anti-inflammatory (M2) to proinflammatory (M1) types during obesity, macrophage regulation of NAFLD/NASH, and the expression of hepatic genes during obesity. In a schematic diagram, they have shown that in obese liver M1-KCs and Ly6C^hi^ macrophages stimulate HSCs that activate myofibroblast leading to fibrosis. The second article by Ray et al. highlights the interaction between the innate/adaptive immune system and the obesity-induced changes in metabolism. They discuss how TNF-α released by M1-macrophages during obesity and lipopolysaccharide released by gut bacteria signal *via* the TNF receptor and toll-like receptors, respectively, and induce inflammation and consequent upregulation of proinflammatory genes. The authors describe polarization of anti-inflammatory adipose tissue M2 macrophage to proinflammatory adipose tissue M1 during obesity. The authors also highlight that polarization of anti-inflammatory M2-KCs to M1-KCs and RHMs including Ly6C^hi^ during obesity results in decreased hepatic insulin sensitivity. The third article by Heinrich et al. highlights the role of hepatic CEACAM1 in obesity across multiple species and most notably demonstrates a significant reduction in hepatic CEACAM1 in obese subjects with fatty liver disease. The fourth article by Bandyopadhyay and Mahata underline contributions of two CgA-derived peptides, namely, PST and CST in regulation of obesity and insulin sensitivity. The authors discuss the mechanisms underlying inhibition of glucose-stimulated insulin secretion, hepatic gluconeogenesis, and insulin-stimulated lipid synthesis by PST. Furthermore, they underscore how PST induces inflammation and endoplasmic reticulum stress leading to the development of insulin resistance. CST, on the other hand, decreases hypertension by inhibiting catecholamine secretion and releasing histamine. They underline that CST alleviates adiposity by increasing lipolysis followed by increased β-oxidation of fatty acids. They also emphasize that CgA is proteolytically processed to counterregulatory peptides such as PST and CST for fine tuning and maintenance of metabolic homeostasis. The fifth article by Sinha-Hikim et al. critically reviews the connections of nicotine and high-fat diet (HFD) to NAFLD. Nicotine when combined with an HFD leads to NAFLD through multiple mechanisms, including generation of severe oxidative stress and increased hepatocellular apoptosis as well inducing adipose tissue lipolysis resulting in excess delivery of free fatty acid and perturbation of hepatic lipid homeostasis through inactivation of AMP-activated protein kinase. Evidence also suggests a central role of the gut microbiota in obesity and its related disorders, including NAFLD. The pathogenesis of human NAFLD remains unclear, in particular in the context of its relationship to insulin resistance and visceral obesity. The sixth article by Sinha et al. underscores that skeletal muscle maintenance is a dynamic process and undergoes constant repair and regeneration. However, skeletal muscle regenerative capacity declines in obesity. They focus on obesity-associated changes in inflammation, metabolism, and impaired insulin signaling, which are pathologically dysregulated and ultimately result in a loss of muscle mass and function. The seventh article (original) by Heinrich et al. demonstrates that loss of hepatic CEACAM1 provides a unifying mechanism linking insulin resistance to obesity and NAFLD. The eighth article by Mishra et al. underlines the physiological steps leading to the development of DCM. The early steps include changes in substrate metabolism (abandoning glucose and relying mostly on fatty acids), oxidative and endoplasmic reticulum stress, formation of extracellular matrix proteins, and advanced glycation end products. The late steps embrace steatosis, apoptosis, fibrosis, and remodeling of cardiomyocytes resulting in DCM constituting left ventricular hypertrophy and reduced diastolic function. In a schematic diagram, they have shown how CCL7 released by activated B cells during obesity causes infiltration of monocyte-derived macrophages and subsequent stimulation of mast cells and infiltration of neutrophils. TGF-β secreted by activated monocyte-derived macrophages stimulates myofibroblasts to induce fibrosis. They also emphasize the differential expressions of various miRNAs in diabetic hearts and their roles in cardiac function and metabolism. The ninth article by Webster underlines the mechanisms underlying alternate RNA splicing and their implications in the development of liver, hepatic steatosis, and hepatocellular carcinoma. The author provides a detailed information on alternative splicing in liver and genetic manipulation of RNA-binding proteins *in vivo*. In addition, he has thoroughly described the RNA splicing SR proteins (with long repeats of serine and arginine) and their crucial roles in the development of hypertrophic and dilated cardiomyopathy, liver damages, and secretion of very low-density lipoproteins and triglycerides. The 10th article by Khullar et al. discusses how cumulative interactive effects of genetic and environmental factors result in the development of diabetes. In particular, the authors describe how modifications in histone acetyl transferases and histone deacetylases with consequent change in gene expression cause diabetes-induced microvascular complications.

## Author Contributions

AS-H and SM have made equal contributions.

## Conflict of Interest Statement

The authors declare that the research was conducted in the absence of any commercial or financial relationships that could be construed as a potential conflict of interest.

